# Transcription Factor Forkhead Regulates Expression of Antimicrobial Peptides in the Tobacco Hornworm, *Manduca sexta*

**DOI:** 10.1038/s41598-017-02830-w

**Published:** 2017-06-02

**Authors:** Xue Zhong, Munmun Chowdhury, Chun-Feng Li, Xiao-Qiang Yu

**Affiliations:** 1 Division of Molecular Biology and Biochemistry, School of Biological Sciences, University of Missouri – Kansas City, Kansas City, MO 64110 USA; 2 State Key Laboratory of Silkworm Genome Biology, Southwest University, Chongqing, 400716 China

## Abstract

Antimicrobial peptides (AMPs) play an important role in defense against microbial infections in insects. Expression of AMPs is regulated mainly by NF-κB factors Dorsal, Dif and Relish. Our previous study showed that both NF-κB and GATA-1 factors are required for activation of moricin promoter in the tobacco hornworm, *Manduca sexta*, and a 140-bp region in the moricin promoter contains binding sites for additional transcription factors. In this study, we identified three forkhead (Fkh)-binding sites in the 140-bp region of the moricin promoter and several Fkh-binding sites in the lysozyme promoter, and demonstrated that Fkh-binding sites are required for activation of both moricin and lysozyme promoters by Fkh factors. In addition, we found that Fkh mRNA was undetectable in *Drosophila* S2 cells, and *M. sexta* Fkh (MsFkh) interacted with Relish-Rel-homology domain (RHD) but not with Dorsal-RHD. Dual luciferase assays with moricin mutant promoters showed that co-expression of MsFkh with Relish-RHD did not have an additive effect on the activity of moricin promoter, suggesting that MsFkh and Relish regulate moricin activation independently. Our results suggest that insect AMPs can be activated by Fkh factors under non-infectious conditions, which may be important for protection of insects from microbial infection during molting and metamorphosis.

## Introduction

In insects, innate immunity is the first line of defense against pathogens and it is composed of both humoral and cellular immune responses^1–5^. Synthesis of small cationic antimicrobial peptides (AMPs) in the fat body of insects is an important defense mechanism of humoral immune responses against microbial infection^6–9^. Expression of insect AMPs is regulated mainly by the evolutionarily conserved Toll and immune deficiency (IMD) pathways^10–13^. The Toll pathway defends against Gram-positive bacteria, fungi and viruses^14, 15^, whereas the IMD pathway acts against Gram-negative bacteria^16^. Both the Toll and IMD pathways activate the Rel/NF-κB family of transcription factors, including Dorsal, Dif (Dorsal-related immunity factor)^17^ and Relish^18^. These NF-κB factors contain N-terminal Rel-homology domain (RHD) that is required for DNA binding and dimerization^19^.

**Figure 1:**
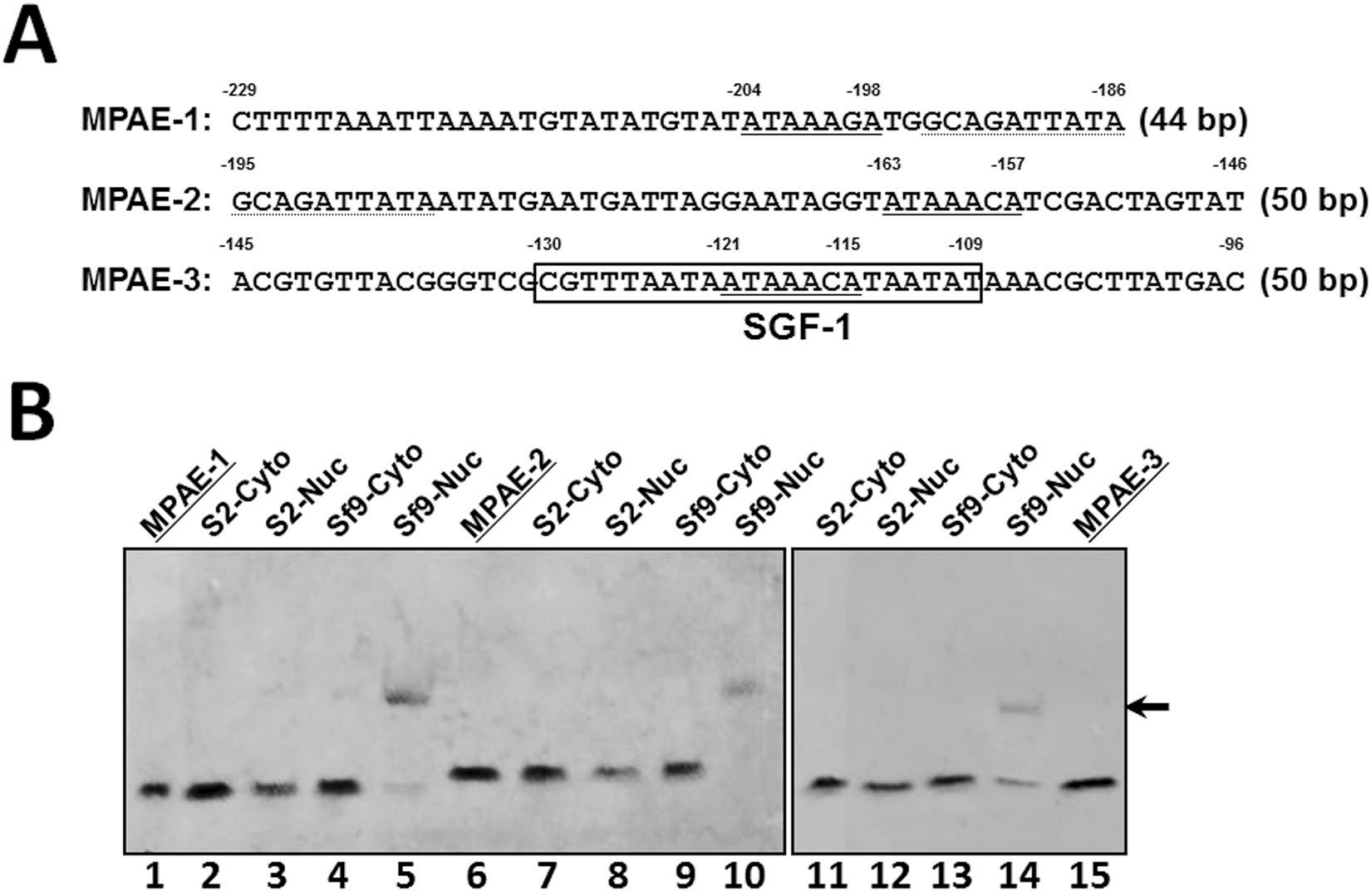
Binding of nuclear proteins from Sf9 cells to MPAE fragments. (**A**) DNA sequences of MPAE-1, −2 and −3 fragments of *M. sexta* moricin promoter. Predicted Fkh-binding sites are underlined and SGF-1 binding site is boxed. The 10-bp overlapping region in MPAE-1 and MPAE-2 fragments was dotted underlined. The numbers above the sequences indicate the positions upstream of the predicted transcription initiation site. (**B**) Electrophoretic mobility shift assay (EMSA). Cytosolic (Cyto) and nuclear (Nuc) proteins were prepared from Sf9 and S2 cells and incubated with biotinylated MPAE-1, −2 or −3 fragments for EMSA assays as described in the Materials and Methods. Lanes 1, 6 and 15 contained only biotinylated MPAE-1, −2 and −3; lanes 2–5, lanes 7–10 and lanes 11–14 contained biotinylated MPAE-1, MPAE-2 and MPAE-3, respectively, with cytosolic or nuclear proteins. Only nuclear proteins from Sf9 cells (Sf9-Nuc) bound to all three biotinylated MPAE fragments and caused mobility shift of the DNA fragments (lanes 5, 10 and 14, arrow).

In our previous study with the moricin promoter in the tobacco hornworm *Manduca sexta*, we found that both NF-κB and GATA-1 binding sites are crucial for activation of moricin promoter, and we also identified a 140-bp region (between −240 and −100 bp) in the moricin promoter, designated as moricin promoter activating element (MPAE), which contains binding sites for additional transcription factors required for activation of moricin promoter^20^. The purpose of this study is to identify the additional transcription factor(s) that can bind to the MPAE region, investigate whether the new factor(s) can also activate other AMP genes, and whether the new factor(s) acts independently or cooperatively with NF-κB factors. Sequence analysis of the 140-bp MPAE region showed a binding site for silk gland factor-1 (SGF-1), a transcription factor in the silk worm *Bombyx mori* that is homologous to *Drosophila melanogaster* forkhead (Fkh). SGF-1 can bind *in vitro* to a *cis*-element located in the promoter region of *sericin-1*, which encodes a silk protein in the middle silk gland (MSG) cells^21–23^. Further analysis showed that there are three Fkh-binding sites in the 140-bp MPAE region (Fig. 1A).

Forkhead transcription factors belong to the Fox (Forkhead box) superfamily proteins^24^. Members of the Fox family proteins consist of a conserved long DNA binding domain connected to a pair of loops or “wings” via a small β-sheet^25^. The long DNA binding domain, also known as winged-helix or forkhead domain, is about 90 residues and composed of three α-helices with a helix–turn–helix core, thus, the Fox family proteins are also known as winged helix transcription factors^26^. Fox proteins have been identified in eukaryotic organisms from yeast to human, and they play important roles in various biological processes, including cellular differentiation, development, metabolism, insulin signaling, immune regulation, cancer development, and aging^27–33^. Forkhead gene A (*FoxA*) was first discovered in *D. melanogaster* since mutation in this gene resulted in fork-headed structure in the embryos with defects in the anterior and posterior gut formation^34^. Later, cDNA encoding hepatocyte nuclear factor 3α (HNF3α), also known as FoxA1, was cloned in rat^35^. The central 90-residue DNA-binding domain (forkhead domain) of *D. melanogaster* FoxA and human FoxA1 shows shockingly high (>85%) identity^36^. Since the discovery of *Drosophila FoxA*, hundreds of Fox genes have been identified in various species. There are at least 4 Fox genes in yeast, 16 in *D. melanogaster*, 15 in *Caenorhabditis elegans*, 44 in mice, and 50 in humans^37–39^. The genome of *Aedes aegypti* contains eighteen loci that encode putative Fox factors and six of them are involved in reproduction^40^. Based on phylogenetic analysis, metazoan Fox family is divided into 19 subfamilies (FoxA-S)^24^. Human Fox subfamilies are classified into two classes based on the sequence homology within and beyond the forkhead domain: Fox A-G, I-L and Q are class 1, Fox H and M-P are class 2 Fox proteins^24^.

In *D. melanogaster*, several Fox transcription factors have been described. Crocodile (FoxC) is involved in early patterning in embryo^41^, Biniou (FoxF) plays a role in visceral mesoderm^42^, sloppy paired 1 & 2 (FoxG) are involved in early embryo segmentation^43, 44^, domina (FoxN) is involved in vitality and fertility^45^, and FoxO regulates the insulin signaling in the brain and fat body^46^. *Drosophila* FoxO is also involved in innate immunity and expression of AMPs^47, 48^. Forkhead (Fkh) is the founding member of the FoxO family and it is activated upon TOR inhibition by rapamycin^48^. However, functions of Fox transcription factors in regulation of AMPs in other insect species have yet to be reported.

In this study, we identified Fkh-binding sites in the promoters of *M. sexta* moricin and lysozyme genes, cloned Fkh genes from both *M. sexta* and *D. melanogaster*, and demonstrated activation of AMP gene promoters by Fkh factors. We also found that Fkh mRNA was undetectable in *Drosophila* S2 cells, which may account for low activity of some AMP promoters in S2 cells. In addition, co-expression study showed that *M. sexta* Fkh (MsFkh) interacted with Relish (Rel2)-RHD, but did not show any interaction with Dorsal-RHD. However, co-expression of MsFkh and MsRelish-RHD did not have an additive effect on the activity of moricin promoter compared to expression of MsFkh or MsRelish-RHD alone, suggesting that MsFkh and MsRelish regulate AMP gene expression independently. Activation of AMPs by Fkh factors under non-infectious conditions is particularly important for insects during molting and metamorphosis to protect them from microbial infections.

## Results

### Binding of nuclear proteins in Sf9 cells to MPAE of *M. sexta* moricin promoter

In our previous study, we identified a 140-bp MPAE region in the *M. sexta* moricin, which was responsible for activation of moricin and *D. melanogaster* drosomycin promoters in *S. frugiperda* Sf9 cells^20^. To identify nuclear factor(s) in Sf9 cells that can bind to the MPAE region, we divided the 140-bp MPAE region into three fragments (MPAE-1 to 3) (Fig. 1A), and performed electrophoretic mobility shift assay (EMSA). EMSA results showed that proteins from nuclear extracts of Sf9 cells, but not S2 cells, bound to all three MPAE fragments (Fig. 1B, lanes 5, 10 and 14, arrow). We then analyzed transcription factor binding sites (AliBaba 2.1 program, http://www.gene-regulation.com) in the three MPAE fragments, and found that there is an SGF-1 binding site in MPAE-3 (Fig. 1A). SGF-1 is a transcription factor in the silkworm *B. mori* that regulates expression of silk protein Sericin-1 in the middle silk gland cells^22, 23^, and it is a homolog of *D. melanogaster* forkhead (Fkh). Fkh is a member of the FoxO family proteins and FoxO binds to the consensus sequence of (T/C)(G/A)AAACAA^49^. We found an ATAAACA sequence in both MPAE-2 and MPAE-3, and an ATAAAGA sequence in MPAE-1 (Fig. 1A). Thus, we speculate that Fkh transcription factor in Sf9 nuclear extracts bound to all three MPAE fragments.

**Figure 2:**
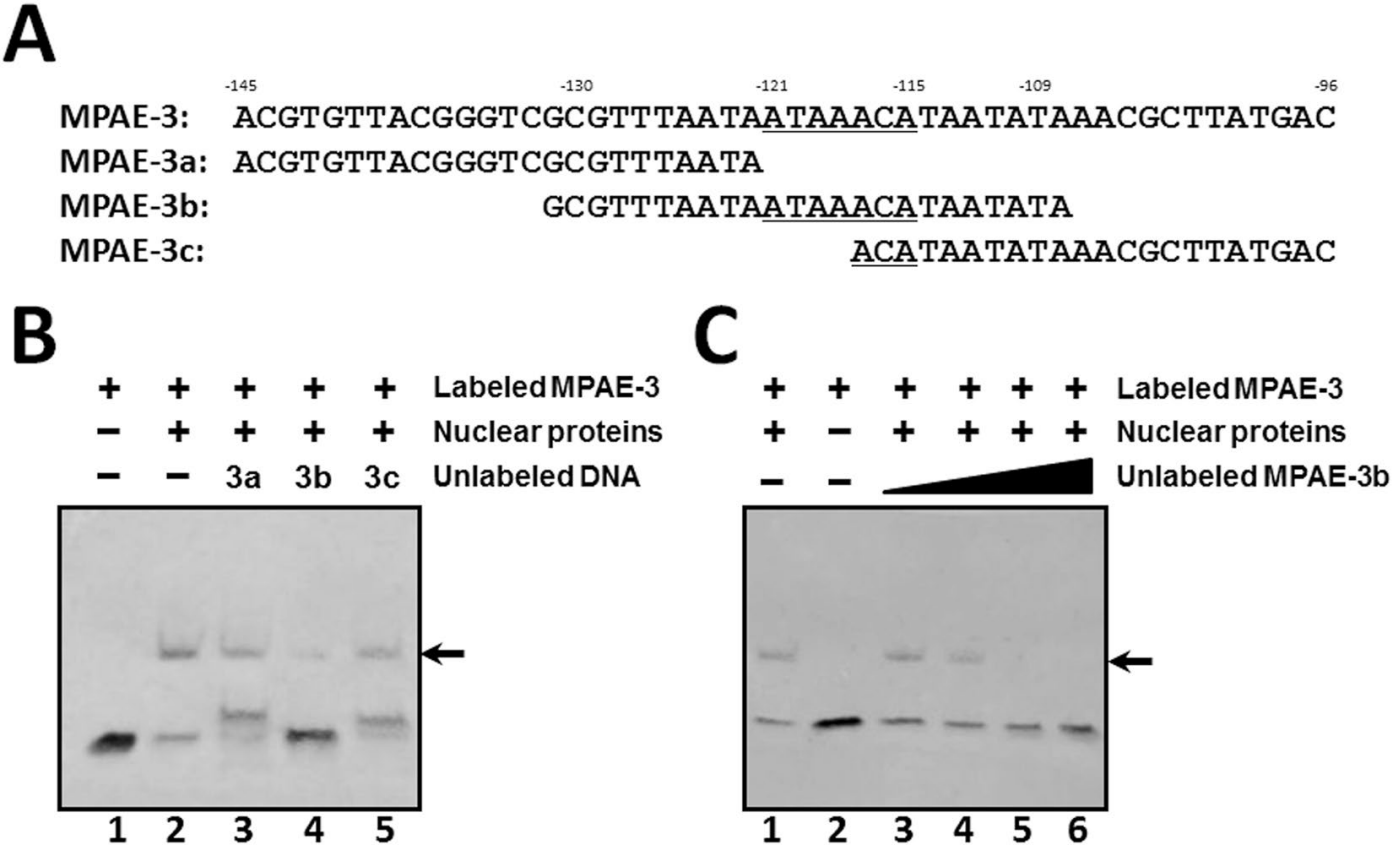
Binding of nuclear proteins from Sf9 cells to Fkh-binding site in MPAE-3. (**A**) DNA sequences of MPAE-3, MPAE-3a, −3b and −3c. Fkh-binding site is underlined. The numbers above the sequence indicate the positions upstream of the predicted transcription initiation site. (**B**, **C**) MPAE-3b competed binding of nuclear proteins to MPAE-3 fragment. Biotinylated MPAE-3 fragment alone (lane 1 in panel B and lane 2 in panel C) or incubated with nuclear proteins from Sf9 cells in the absence (lane 2 in panel B and lane 1 in panel C) or presence of excess (200-fold) unlabeled MPAE-3a, −3b, or −3c (lanes 3–5 in panel B), or in the presence of increasing amount of excess (1-, 10-, 100- and 200-fold) unlabeled MPAE-3b (lanes 3–6 in panel C), and EMSA assays were performed the same as in Fig. 1. Arrows indicated the mobility-shifted bands.

To further determine DNA binding motif in the MPAE region, we selected MPAE-3 fragment and divided it into three smaller fragments (22–24 bp) with 10-bp overlapping region (MPAE-3a to 3c) (Fig. 2A) for competitive EMSA assays. The results showed that only MPAE-3b fragment, which contains the ATAAACA sequence, competed with binding of the labeled MPAE-3 to nuclear proteins of Sf9 cells and the competition by MPAE-3b was dose-dependent (Fig. 2B, lane 4 and Fig. 2C, lanes 3–6, arrows). These results suggest that Fkh transcription factor in Sf9 cells binds to the ATAAACA sequence in the moricin promoter.

### Activation of *M. sexta* moricin and lysozyme promoters by Fkh factors

**Figure 3:**
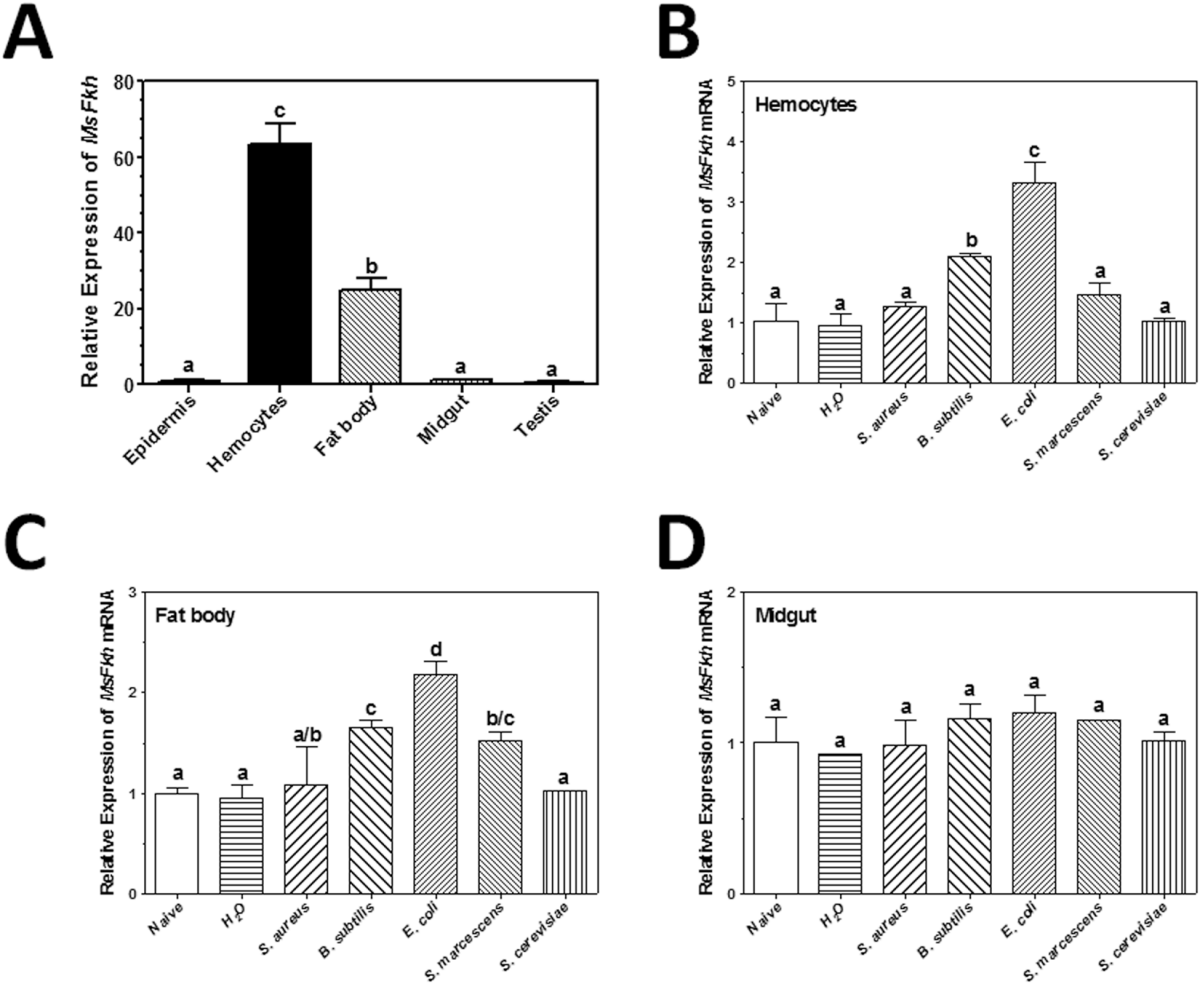
Tissue distribution and induced expression of *M. sexta* Fkh (*MsFkh*) mRNA. Fat body, hemocytes, midgut, epidermis and testis were collected from *M. sexta* naïve fifth-instar larvae (**A**). Hemocytes (**B**), fat body (**C**) and midgut (**D**) were also collected from *M. sexta* larvae at 24 h post-injection of bacteria or yeast for preparation of total RNAs and cDNAs as described in the Materials and Methods. Real-time PCRs were performed with these RNA samples using *M. sexta* ribosomal protein S3 (*rpS3*) gene as an internal standard. Expression of *MsFkh* transcript in the epidermis of naïve larvae (**A**), or in the hemocytes (**B**), fat body (**C**) and midgut (**D**) of naïve larvae was arbitrarily set as 1. Bars represent the mean of three independent measurements ± SEM. Comparing different tissues (**A**) or different injection conditions (**B**–**D**), identical letters are not significant difference (p > 0.05) while different letters indicate significant difference (p < 0.05).

To investigate functions of *M. sexta* Fkh (MsFkh), we first cloned *MsFkh* cDNA and analyzed tissue distribution and induced expression of *MsFkh* mRNA. Real-time PCR results showed that *MsFkh* mRNA was highly expressed in the hemocytes of the fifth- instar *M. sexta* naïve larvae compared to fat body, midgut and other tissues (Fig. 3A), and expression of *MsFkh* transcript was induced in hemocytes (Fig. 3B) and fat body (Fig. 3C) by some bacteria (*B. subtilis* and *E. coli*), but was not induced in the midgut by any of the microorganisms tested (Fig. 3D).

**Figure 4:**
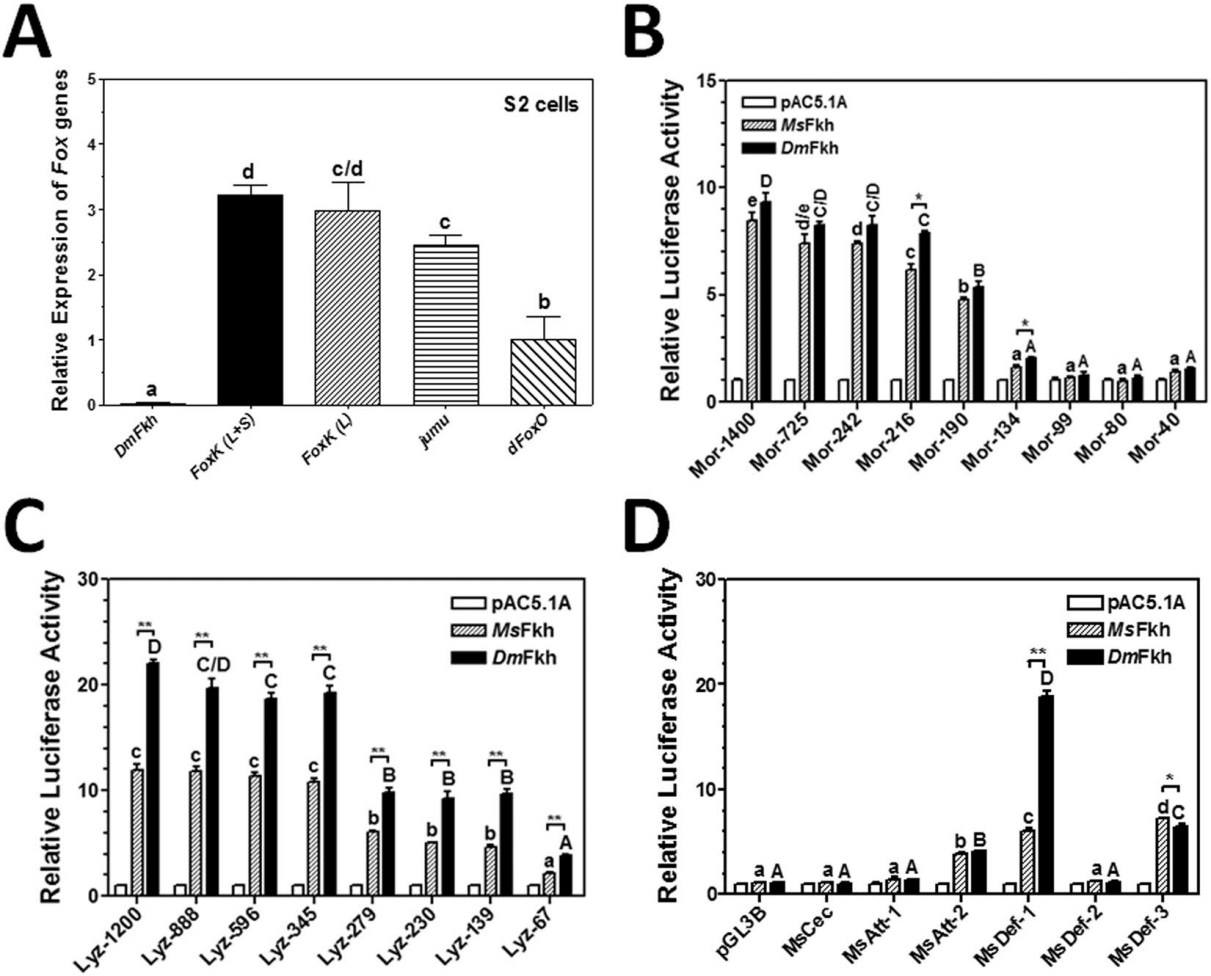
Activation of AMP gene promoters by *M. sexta* and *D. melanogaster* Fkh factors. (**A**) Real-time PCR analysis of transcripts of *Fkh* (*DmFkh*), *FoxK* long and short isoforms (*FoxK* (L + S)), *FoxK* long isoform (*FoxK* (L)), *jumu* and *FoxO* (*dFoxO*) in S2 cells. *Drosophila* ribosomal protein 49 (*rp49*) gene was used as an internal standard. (**B**–**D**) Activation of AMP promoters by overexpression of MsFkh and DmFkh. The relative luciferase activities of truncated moricin promoters (**B**), truncated lysozyme promoters (**C**), and different *M. sexta* AMP gene promoters (**D**) activated by recombinant MsFkh or DmFkh in S2 cells were determined by Dual-Luciferase® Reporter Assay System as described in the Materials and Methods. Bars represent the mean of three independent measurements ± SEM. For transcription levels of Fox genes in S2 cells (**A**), or relative luciferase activity (**B**–**D**) among different promoters activated by one transcription factor (comparing striped bars or solid bars), identical letters are not significant difference (p > 0.05) while different letters indicate significant difference (p < 0.05). For the activity of the same promoter stimulated by different transcription factors (between MsFkh and DmFkh), the significance of difference was also determined by an unpaired t-test (*p < 0.05; **p < 0.01). MsCec, MsAtt-1, MsAtt-2, MsDef-1, MsDef-2 and MsDef-3 are *M. sexta* cecropin, attacin-1, attacin-2, defensin-1, defensin-2 and defensin-3 promoters (See Fig. S1 for the promoter sequences).

Since MPAE did not bind to nuclear proteins in S2 cells (Fig. 1B), and *Drosophila Fkh* (*DmFkh*, CG10002) is not expressed in S2 cells (Flybase), we performed real-time PCR to determined transcripts of several *Drosophila* Fox genes in S2 cells, including *DmFkh*, *FoxK* long and short isoforms, *jumeau* (*jumu*) and *dFoxO*. The results showed that mRNAs of *FoxK*, *jumu* and *dFoxO*, but not *DmFkh*, were detected in S2 cells, with higher transcript level of *FoxK* and *jumu* than *dFoxO* (Fig. 4A), confirming that *DmFkh* was not expressed (or was expressed at an undetectable level) in S2 cells. To test whether overexpression of DmFkh in S2 cells can activate expression of AMP genes, *DmFkh* was also cloned.

To investigate activation of *M. sexta* moricin and lysozyme promoters by forkhead factors, MsFkh and DmFkh were over-expressed in S2 cells, and dual luciferase assays were performed with several truncated moricin and lysozyme promoters. MsFkh is 355 residues long with a theoretical molecular weight of 39.5 kDa and *p*I of 9, while DmFkh (Forkhead isoform A) is 510 residues with molecular weight of 54.3 kDa and *p*I of 8.7. The full length MsFkh shows 96% identity in amino acid sequence to *B. mori* SGF-1 (Genbank accession no. NP_001037329.1), 95% identity to *Helicoverpa armigera* (Genbank accession no. AAW56613.1) and *Spodoptera exigua* (Genbank accession no. ACA30303.1) forkhead domain transcription factors, but only 42% identity to the full length DmFkh. However, the forkhead domains of MsFkh and DmFkh proteins share 98% identity. Dual luciferase results showed that both MsFkh and DmFkh activated *M. sexta* moricin, lysozyme, defensin-1, defensin-3 and attacin-2 promoters, but did not activate *M. sexta* cecropin, attacin-1, or defensin-2 promoter (Fig. 4B–D). DmFkh stimulated the activity of AMP promoters to a similarly high level as or to a higher level than MsFkh. Comparing different truncated promoters of *M. sexta* moricin and lysozyme promoters, the 1.4-kb moricin promoter (Mor-1400) was activated by Fkh factors to a similarly high level as the 242-bp truncated moricin promoter (Mor-242), but further truncation of the 242-bp moricin promoter significantly decreased the activity of the truncated promoters (Mor-190, Mor-134, Mor-99 and Mor-80) (Fig. 4B), suggesting that the Fkh-binding sites are within the MPAE region. Similarly, the 1.2-kb lysozyme promoter (Lyz-1200) and the 345-bp truncated promoter (Lyz-345) showed a similarly high activity in S2 cells after overexpression of MsFkh or DmFkh, and further truncation of the Lyz-345 promoter significantly decreased the activity of the truncated lysozyme promoters Lyz-279, Lyz-230, Lyz-139 and Lyz-67 (Fig. 4C), indicating that the Fkh-binding sites are within the 345-bp region of the lysozyme promoter.

### Identification of Fkh-binding sites in *M. sexta* moricin and lysozyme promoters

**Figure 5:**
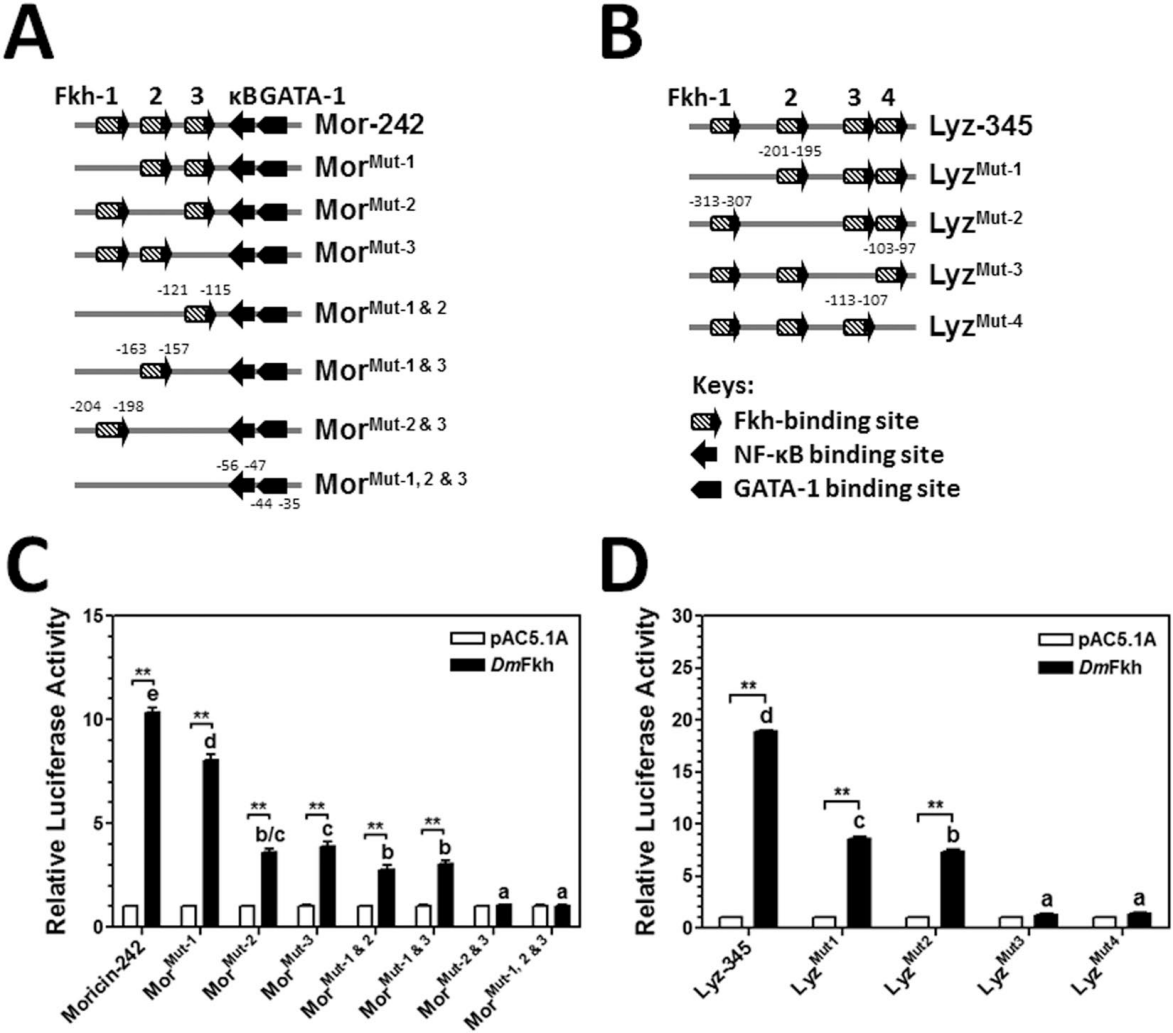
Identification of active Fkh-binding sites in *M. sexta* moricin and lysozyme promoters. (**A**, **B**) Schematic diagrams of the truncated Mor-242 (**A**) and Lyz-345 (**B**) promoters. Fkh-1, 2, 3, 4 indicate the predicted Fkh-binding sites 1, 2, 3, 4; κB and GATA-1 indicate the NF-κB and GATA-1 binding sites in the moricin promoter. Mut-1, −2, −3, −4 indicate the mutation of Fkh-binding sites 1, 2, 3, 4; Mut-1 & 2, Mut-1 & 3, Mut-2 & 3, and Mut-1, 2 & 3 indicate the mutations of Fkh-binding sites 1 and 2, 1 and 3, 2 and 3, as well as 1, 2 and 3, respectively. (**C, D**) Activation of Mor-242 and Lyz-345 promoters by recombinant DmFkh. The relative luciferase activities of Mor-242 and its Fkh-binding site mutant promoters (**C**), Lyz-345 and its Fkh-binding site mutant promoters (**D**) activated by recombinant DmFkh in S2 cells were determined by Dual-Luciferase® Reporter Assay System as described in the Materials and Methods. Bars represent the mean of three independent measurements ± SEM. For the relative luciferase activity among different promoters activated by DmFkh (comparing the solid bars), identical letters are not significant difference (p > 0.05) while different letters indicate significant difference (p < 0.05). For the activity of the same promoter after overexpression of DmFkh (comparing the solid and open bars for each promoter), the significance of difference was also determined by an unpaired t-test (*p < 0.05; **p < 0.01).

To identify Fkh-binding sites in the MPAE region of *M. sexta* moricin promoter and the 345-bp region of lysozyme promoter that are responsible for activation by Fkh factors, we first analyzed the Fkh-binding sites in the 140-bp MPAE and the 345-bp region of lysozyme promoter. Three and four Fkh-binding sites with the core sequence of AAACA were predicted within the 140 bp MPAE of moricin and 345 bp of lysozyme promoters, respectively (Figs. 1A, 5A,B and S1). To determine the active Fkh-binding sites in moricin and lysozyme promoters, the truncated Mor-242 and Lyz-345 promoters were selected for mutation of each Fkh-binding site for dual luciferase assays (Fig. 5A,B). Mutation of Fkh-binding site 2 or 3 alone in the Mor-242 promoter significantly decreased the activity of the mutant promoter by more than 60% compared to Mor-242 promoter, and mutation of both sites 2 and 3 together completely abolished activation of the mutant Mor-242 promoter by DmFkh (Fig. 5C). Mutation of Fkh-binding site 1 decreased the activity of the mutant promoter by ~20% compared to Mor-242 promoter, while mutation of both sites 1 and 2 or sites 1 and 3 together did not significantly decrease the activity further compared to mutation of site 2 or site 3 alone (Fig. 5C). These results suggest that Fkh-binding sites 2 and 3 in the MPAE region of moricin promoter play an equally important role in activation of moricin, while Fkh-binding site 1 may also contribute to activation of moricin. Similarly, mutation of the Fkh-binding site 1 or 2 alone in the Lyz-345 promoter significantly decreased the activity by more than 50%, and mutation of the Fkh-binding site 3 or 4 alone completely abolished DmFkh-activated Lyz-345 promoter activity (Fig. 5D), suggesting that Fkh-binding sites 3 and 4 play an important role in activation of lysozyme by Fkh factor whereas Fkh-binding sites 1 and 2 also contribute to regulation of lysozyme.

### Interaction of *M. sexta* Fkh with Relish-RHD

**Figure 6:**
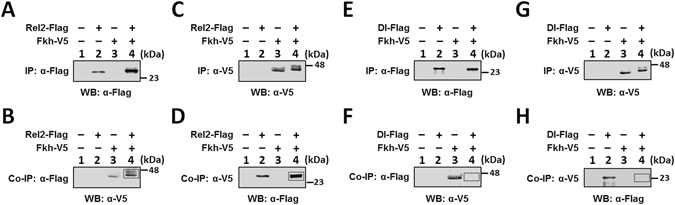
Interaction of *M. sexta* Fkh with Relish-RHD. Recombinant V5-tagged MsFkh, Flag-tagged MsRelish-RHD (MsRel2-RHD) and Flag-tagged MsDorsal-RHD (MsDl-RHD) were expressed in S2 cells separately, and cell lysates from two samples were mixed for co-immunoprecipitation (Co-IP) assays as described in the Materials and Methods. Immunoprecipitated (IP) proteins or Co-IP proteins were detected by immunoblotting using anti-Flag or anti-V5 monoclonal antibody as the primary antibody. Lanes 1–3 were cell lysates (protein inputs) from control S2 cells (lane 1), S2 cells overexpressing Flag-tagged MsRel2-RHD or Flag-tagged MsDl-RHD (lane 2), and S2 cells overexpressing V5-tagged MsFkh (lane 3), and lane 4 was IP or Co-IP proteins. V5-tagged MsFkh was co-immunoprecipitated with Flag-tagged MsRel2-RHD (**B** and **D**, lane 4, boxed bands), but was not co-immunoprecipitated with Flag-tagged MsDl-RHD (**F** and **H**, lane 4, boxes).

In the Mor-242 promoter, an NF-κB and a GATA-1 binding sites, which were both required for activation of moricin by immune signaling pathway^20^, were still present near the transcription initiation site (Fig. 5A), while in the Lyz-345 promoter, the NF-κB site was absent (the only NF-κB site in the 1.2-kb lysozyme promoter was between −1191 and −1182 bp, Fig. S1). In order to test whether NF-κB and Fkh factors regulate AMP genes independently or cooperatively, we first determined interaction of Fkh factor with NF-κB factors Dorsal and/or Relish (Rel2). Co-immunoprecipitation (Co-IP) assays showed that V5-tagged MsFkh co-precipitated with Flag-tagged *M. sexta* Rel2-RHD (Fig. 6B, D, lane 4), but did not co-precipitate with Flag-tagged Dorsal-RHD (Dl-RHD) (Fig. 6F, H, lane 4), suggesting that MsFkh interacts with MsRelish but did not interact with MsDorsal.

### Regulation of AMP genes independently by *M. sexta* Fkh and Relish

**Figure 7:**
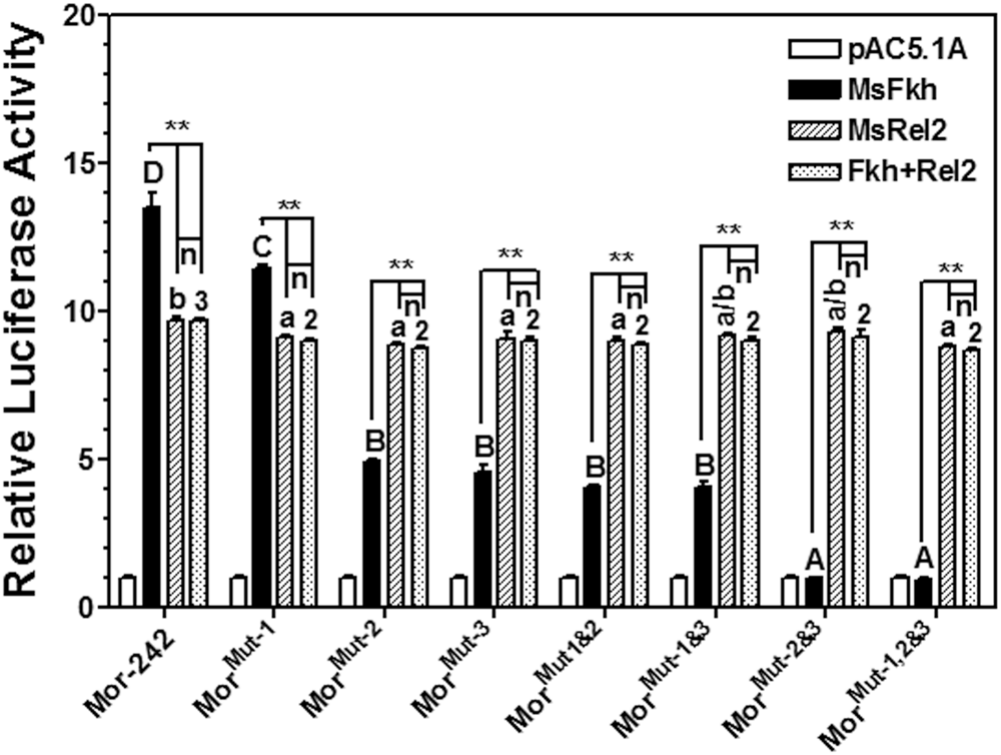
Activation of moricin promoters by MsFkh and MsRel2-RHD independently. The relative luciferase activities of the truncated Mor-242 and its Fkh-binding site mutant promoters activated by recombinant MsFkh or MsRel2-RHD alone, or by co-expression of MsFkh and MsRel2-RHD in S2 cells were determined by Dual-Luciferase® Reporter Assay System as described in the Materials and Methods. Bars represent the mean of three independent measurements ± SEM. For the activity among different promoters activated by transcription factors (comparing solid bars by MsFkh, stripe bars by MsRel2-RHD, or dotted bars by MsFkh/MsRel2-RHD across the promoters), identical letters (capital letters for solid bars and small letters for stripe bars) or identical numerical numbers (dotted bars) are not significant difference (p > 0.05) while different letters or different numerical numbers indicate significant difference (p < 0.05). Comparing the activity of the same promoter stimulated by different transcription factors (between solid and stripe bars, solid and dotted bars, as well as stripe and dotted bars for each promoter), the significance of difference was also determined by an unpaired t-test (*p < 0.05; **p < 0.01), and “n” indicates not significant.

To determine whether interaction of MsFkh with MsRelish has an impact on regulation of moricin promoter by MsFkh and MsRelish, we used Mor-242 and its seven Fkh mutant promoters (Fig. 5A) for the dual luciferase assays. For MsFkh-stimulated activity (Fig. 7, solid bars), mutations of individual Fkh-binding sites alone (site 1, 2 or 3) or combination of Fkh-binding sites (sites 1 and 2, 1 and 3, 2 and 3, or all three sites) significantly decreased the activity of Fkh mutant promoters, a result similar to that in Fig. 5C, indicating that Fkh-binding sites indeed play a role in activation of moricin promoter by Fkh factor. Co-expression of MsFkh and MsRel2-RHD had similar effect as overexpression of MsRel2-RHD alone (Fig. 7, comparing stripe bars and dotted bars across different promoters, as well as between the stripe and dotted bars in each promoter), suggesting that presence/absence of MsFkh and Fkh-binding sites does not have an impact on activation of moricin promoter by MsRel2-RHD, and the overall activity of these moricin promoters is due to MsRel2-RHD binding to NF-κB site in the moricin promoter. Co-expression of MsFkh and MsRel2-RHD activated the activity of moricin promoters to either significantly higher or significantly lower level than that activated by overexpression of MsFkh alone, depending on the presence/absence of Fkh-binding sites (Fig. 7, comparing the solid bars with dotted bars in each promoter), further confirming that when MsFkh and MsRel2-RHD are expressed together, MsRel2-RHD tends to bind NF-κB sites regardless of MsFkh and Fkh-binding sites. Together, these results suggest that even though MsFkh can interact with MsRelish, formation of MsRelish homodimers may be predominant, and MsFkh and MsRelish regulate moricin promoter activation independently.

## Discussion

Synthesis of AMPs is a major defense mechanism against infection in insects^50–54^, and expression of AMPs is regulated by the Toll and IMD pathways via activation of NF-κB transcription factors Dorsal, Dif and Relish^55, 56^. Other proteins and factors that can modulate the Toll and/or IMD pathways have been identified. For example, a Zn finger homeodomain 1 (zfh1) transcription factor has been reported as a negative regulator of *Drosophila* IMD pathway downstream of, or parallel to Relish^57^; Dorsal interacting protein 3 (Dip3) can bind to the RHD of Dorsal and Relish via its BESS domain, and it functions in both dorsoventral patterning and immune response^58^. In *Drosophila*, it has been reported that activation of AMPs can be achieved by the transcription factor FoxO independent of the immune signaling pathways^47^, and induction of two AMPs, diptericin and metchnikowin, after downregulation of TOR by rapamycin is regulated by the transcription factor forkhead (Fkh)^48^. FoxO is an important regulator of stress, metabolism and aging^59–62^, and a key transcription factor in the insulin signaling pathway^63, 64^; whereas Fkh is the founding member of the FoxO family. However, very little is known about regulation of AMPs in other insect species by the FoxO family transcription factors.

Insects can synthesize a variety of AMPs, some AMPs are common to most insects, whereas some other AMPs are found only in certain insect species^52^. For example, moricin and gloverin have been identified only in the lepidopteran insects. We have previously shown that *M. sexta* moricin is regulated by NF-κB factor Relish and GATA-1 factor^20, 65^, and it can also be activated by unidentified nuclear factor(s) that bind to the MPAE region of moricin promoter^20^. In this study, we identified three Fkh-binding sites in the MPAE region and demonstrated that Fkh-binding sites 2 and 3 played an equally important role in activation of moricin promoter by Fkh factor. We also identified four Fkh-binding sites in *M. sexta* lysozyme promoter and all four Fkh-binding sites are important for activation of lysozyme promoter by Fkh factor. Since the consensus sequence for FoxO is about 8 bp [(T/C)(G/A)AAACAA]^49^ with a core sequence of AAACA, there are many predicted/potential Fkh-binding sites in promoters. Thus, we used truncated promoters first to narrow the length of promoters and then focused on potential active Fkh-binding sites in the moricin and lysozyme promoters with the core sequence of AAACA, as the core sequence may be crucial for binding of Fkh factor. Among the three and four Fkh-binding sites in the Mor-242 and Lyz-345 truncated promoters, all six Fkh-binding sites but the binding site 1 in the Mor-242 contains the AAACA core sequence (Figs. 1A and 5B). Indeed, mutation of Fkh-binding site 1 (with the core sequence of AAAGA) did not decrease the activity of Mor-242 promoter as significantly as did mutation of Fkh-binding site 2 or 3, suggesting that the core sequence of AAACA is crucial for activation of genes by Fkh factor. In addition to moricin and lysozyme promoters, Fkh factor also activated *M. sexta* defensin-1, defensin-3 and attacin-2 promoters. To our knowledge, this is the first report about activation of AMPs by Fkh factors in a lepidopteran insect. Activation of AMPs by Fkh factor under non-infectious conditions may be important for insects during molting and metamorphosis, since insects at these particular developmental stages are vulnerable to infection, and induced expression of AMPs by Fkh factor may protect insects from microbial infection. We observed that DmFkh stimulated the activity of some AMP promoters to a higher level than MsFkh did in S2 cells. This may be because MsFkh is not completely compatible in *Drosophila* S2 cells or the longer DmFkh (510 residues) may contain some other domains/motifs that can help Fkh to activate AMP gene promoters.

Different transcription factors may regulate gene expression independently or cooperatively. For *M. sexta* moricin, we previously found that both NF-κB and GATA-1 factors are required for activation of moricin promoter^20^. We also found that *M. sexta* Dorsal can interact with Relish (Rel2), and Dorsal/Relish heterodimers serve as negative regulators to prevent over-activation of *M. sexta* AMP genes^65^. We found that MsFkh interacted with MsRel2-RHD. In the Mor-242 promoter, which contains both NF-κB and Fkh binding sites, overexpression of MsFkh factor alone activated the promoter activity to a significantly higher level compared to overexpression of MsRel2-RHD alone, suggesting that Fkh factor plays an important role in activation of moricin under non-infectious conditions. In the mutant Mor-242 promoters in which the Fkh-binding sites were mutated, co-expression of MsFkh and MsRel2-RHD activated the activity of Fkh-binding site mutant promoters to a similar high level as that activated by MsRel2-RHD alone, indicating that MsFkh and MsRel2 regulate moricin activation independently. This result also suggests that although MsFkh can interact with MsRelish to form heterodimers, homodimers of MsRelish may be predominant. This may be because the distance between Fkh and NF-κB binding sites in the moricin promoter (~58 bp between NF-κB site and Fkh-binding site 3, Fig. 5A) is too far away for the two factors to form heterodimers. For NF-κB and GATA-1 sites in the moricin promoter, the two sites are separated by only 2 bp (Fig. 5A), and we showed that both NF-κB and GATA-1 sites are required for activation of moricin promoter^20^.

We also confirmed that *Fkh* mRNA was undetectable in *Drosophila* S2 cells, but the transcripts for *FoxK* (long and short isoforms), *jumu* and *dFoxO* were detected. Nuclear proteins from S2 cells did not bind to MPAE region, further supporting that *DmFkh* is not expressed (or was expressed at an undetectable level) in S2 cells. This information is important when performing promoter reporter assays for Fkh factor in S2 cells, as stress conditions may not activate the promoters in S2 cells due to lack of endogenous Fkh factor. In future, we will investigate activation of AMPs under non-infectious conditions via Fkh factor and maybe other members of the FoxO family and whether enhanced expression of AMPs by FoxO family members can protect insects from microbial infection during molting and/or metamorphosis.

## Methods

### *Manduca sexta* and insect cell lines

We purchased *M. sexta* eggs from Carolina Biological Supplies (Burlington, NC, USA) and reared larvae to fifth-instar on an artificial diet at 25 °C^66^ for all the experiments. *D. melanogaster* Schneider S2 cells were obtained from American Type Culture Collection (ATCC), and *Spodoptera frugiperda* Sf9 cells were from Invitrogen (12552-014, Invitrogen). Cells were maintained at 27 °C in Insect Cell Culture Media (SH30610.02, Hyclone) supplemented with 1% penicillin-streptomycin solution (G6784, Sigma-Aldrich) and 10% heat-inactivated fetal bovine serum (#10082063, Invitrogen).

### Electrophoretic mobility shift assay (EMSA)

Cytosolic and nuclear proteins were isolated from S2 and Sf9 cells using the Nuclear Extraction Kit (2900, EMD Millipore) following the manufacturer’s instructions. Briefly, S2 and Sf9 cells with 70–80% confluency were collected (2 × 10^8^ cells) and homogenized in 500 μl of 1 × Cytoplasmic Lysis Buffer with 27-gauge needle and centrifuged at 8,000 × g for 20 min at 4 °C. The supernatants containing the cytosolic proteins were transferred to fresh tubes and stored at −80 °C for later use, whereas the pellets were resuspended in 100 μl of ice-cold Nuclear Extraction Buffer containing 0.5 mM DTT and 1/1000 Protease Inhibitor Cocktail. The nuclei were disrupted using 27-gauge needle, the mixture was incubated at 4 °C for 60 min with gentle agitation, and then centrifuged at 16,000 × g for 5 min at 4 °C. These supernatants containing nuclear proteins were removed to fresh tubes and stored at −80 °C for later use.

Electrophoretic mobility shift assay (EMSA) was performed using the LightShift^®^ Chemiluminescent EMSA Kit (20148, Thermo Scientific) following the manufacturer’s instructions. Genomic DNA fragments from the 140-bp MPAE region of moricin promoter (MPAE-1, −2 and −3, as well as MPAE-3a, −3b and −3c) were synthesized with or without covalently linked biotins by Integrated DNA Technologies (IDT) (San Diego, CA). EMSA was performed in 20 μl reactions containing 2 μl of biotinylated DNA (20 fmole) with 3 μg of cytosolic or nuclear proteins from S2 or Sf9 cells, in the absence or presence of unlabeled DNAs (200-fold, or from 1-, 10-, 100- to 200-fold molar excess of the biotinylated DNA). The mixtures were incubated at room temperature for 30 min, separated on 6% polyacrylamide gel (EC6365BOX, Invitrogen) pre-run in 0.5 × TBE at 100 V for 60 min. The gels were then transferred to nylon membrane pre-soaked in 0.5 × TBE at 380 mA for 30 min in ice-cold 0.5 × TBE, and the membranes were crosslinked for 1 min using a UV-light crosslinking instrument. The biotinylated DNA was detected by Streptavidin-Horseradish Peroxidase conjugated anti-mouse antibody (SC-2005, Santa Cruz Biotechnology, 1:10,000) by chemiluminescence using ECL Chemiluminescence Detection Kit (RPN2134, GE Healthcare), and the membranes were exposed to films and scanned by Typhoon FLA7000 (GE healthcare).

### Analyses of transcripts of *M. sexta* forkhead (MsFkh) in larvae and *D. melanogaster* Fox genes in S2 cells

There are two cDNA sequences for *D. melanogaster* forkhead (transcript variant A) (DmFkh) in the database (Genbank accession numbers: J03177.1 and NM_079818.3) with identical open reading frame (ORF) of 1530 bp, which encodes 510 amino acid of DmFkh. To identify *M. sexta* forkhead, we used the ORF of DmFkh to blast the *M. sexta* database (http://agripestbase.org/manduca/?q=blast) and obtained one sequence [Msex2.13928-RA, scaffold01234:21957-23009(-)], which is significantly similar to DmFkh (E-value = e^−22^). This *M. sexta* cDNA sequence contains an ORF of 1065 bp, encoding a protein of 355 amino acids with a forkhead domain. We named this protein *M. sexta* Fkh (MsFkh).

To determine tissue distribution of *MsFkh* mRNA, day 2 fifth-instar *M. sexta* naïve larvae were dissected. Hemocytes, fat body, midgut, epidermis and testis were collected and washed 3 times in anti-coagulant (AC) saline (4 mM NaCl, 40 mM KCl, 8 mM EDTA, 9.5 mM citric acid-monohydrate, 27 mM sodium citrate, 5% sucrose, 0.1% polyvinylpyrrolidone, 1.7 mM PIPES). Total RNAs were extracted from these tissues with TRIzol® Reagent (T9424, Sigma-Aldrich) and cDNAs were prepared from total RNAs (1 μg for each sample) using moloney murine leukemia virus (M-MLV) reverse transcriptase (M1701, Promega) with an anchor-oligo(dT)_18_ primer following the manufacturer’s instructions as described previously^65^.

To determine induced expression of *MsFkh* transcript in *M. sexta* larvae, day 2 fifth-instar naïve larvae were injected with heat-killed *Staphylococcus aureus*, *Bacillus subtilis*, *Escherichia coli* strain XL1-blue, *Serratia marcescens* (each at 5 × 10^7^ cells/larva), or *Saccharomyces cerevisiae* (10^7^ cells/larva), or with water as a control. Hemocytes, fat body and midgut were collected separately at 24 h post-injection for total RNA extraction and cDNA preparation as described previously^67^. Total RNA and cDNA were also prepared from *Drosophila* S2 cells. Briefly, S2 cells (5 × 10^6^ cells) were collected in 1 ml of TRIzol® Reagent (T9424, Sigma-Aldrich) and homogenized using hand held pestle and mixer (Argos Technologies, Elgin, IL). Then, 200 μl of chloroform were added, and the mixture was vortexed and centrifuged at 12000 × g at 4 °C for 15 min. The top aqueous phase (200 μl) was transferred to fresh Eppendorf tubes and isopropanol (500 μl) was added. RNA was precipitated by centrifugation at 12,000 × g at 4 °C for 10 min. The RNA pellets were washed with chilled 70% ethanol, air dried and re-suspended in 50 μl of nuclease free water and stored at −80 °C for later use.

Real-time PCR was performed for *MsFkh* (primers MsFkh-N and MsFkh-C, amplicon size of 151 bp) in different tissues of *M. sexta* larvae and several *Drosophila* Fox genes in S2 cells, including *DmFkh* (Genbank accession no. NP_524542.1) (primers DmFkh-N and DmFkh-C, 150 bp), forkhead box K (*FoxK*) long and short isoforms (Genbank accession no. AY787838) (primers DmFoxK-(L + S)-N and DmFoxK-(L + S)-R for both isoforms, primers DmFoxK-(L)-N and DmFoxK-(L)-R for long isoform only, amplicon sizes of 150 bp), which are also known as forkhead transcription factor long isoform (mnf-l) and short isoform (mnf-s), *jumu* (Genbank accession no. NM_079578.3) (primers DmJumu-N and DmJumu-R, 150 bp) and *dFoxO* (Genbank accession no. NM_206483.3) (primers dFoxO-N and dFoxO-R, 150 bp), in 20 μl reactions containing 10 μl 2 × SYBR^®^ GreenER^TM^ qPCR SuperMix Universal (No. 204141, Qiagen), 4 μl H_2_O, 4 μl diluted cDNA template, and 1 μl forward and reverse primers (10 pmol each), and *M. sexta* ribosomal protein S3 (*rpS3*) (primers rpS3-N and rpS3-C, 150 bp) or *D. melanogaster* ribosomal protein 49 (*rp49*) (primers rp49-N and rp49-C, 150 bp) gene was used as an internal standard to normalize the amount of RNA template. Real-time PCR program was 50 °C for 2 min, 95 °C for 10 min, followed by 40 cycles of 95 °C for 15 s, 60 °C for 1 min and the dissociation curve analysis. Data from three replicas of each sample were analyzed by the ABI 7500 SDS software (Applied Biosystems) using a comparative method (**2** ^−∆∆**CT**^)^68, 69^. These experiments were repeated with three different biological samples.

### Construction of recombinant MsFkh and DmFkh pAC5.1/V5-His A expression vectors

Recombinant MsRelish-RHD (MsRel2-RHD) and MsDorsal-RHD (MsDl-RHD) with a Flag-tag in pAC5.1/V5-His A expression vectors were already constructed as described previously^65^. To construct V5-tagged Fkh into pAC5.1/V5-His A expression vector, cDNA fragments encoding MsFkh (residues 1–355) and DmFkh (residues 1–510) were amplified by PCR using forward and reverse primers (Table S1). Forward primers for MsFkh and DmFkh (MsFkh-F-*Kpn* I and DmFkh-F-*Kpn* I) contain a *Kpn* I site and the reverse primers (MsFkh-R-*Not* I and DmFkh-R-*Not* I) contain a *Not* I site in-frame fused to V5 tag and stop codon of pAC5.1/V5-His A expression vector. PCR reactions were performed with the following conditions: 94 °C for 3 min, 35 cycles of 94 °C for 30 s, Tm-5 °C for 30 s, 72 °C for 30 s to 4 min, followed by a final extension at 72 °C for 10 min. The PCR products were recovered by agarose gel electrophoresis-Wizard® SV Gel and PCR Clean-Up System (A9285, Promega) and subcloned into T-Easy vectors (A1360, Promega). Recombinant T-vectors were purified using PureYield™ Plasmid Miniprep System (A1222, Promega) according to the manufacturer’s instruction and digested with *Kpn* I/*Not* I restriction enzymes, and DNA fragments were recovered and inserted into *Kpn* I/*Not* I digested pAC5.1/V5-His A expression vector using T4 DNA ligase (M0202L, NEB). Recombinant plasmids were then purified and sequenced by an Applied Biosystems 3730 DNA Analyzer in the DNA Sequencing and Genotyping Facility at University of Missouri – Kansas City, and used to transfect S2 cells.

### Construction of luciferase reporter plasmids

The moricin and lysozyme truncated promoters were constructed as described previously^20, 65^. To construct Fkh-binding site mutation promoters, site-directed mutagenesis was performed using the truncated *M. sexta* moricin-242 (242 bp) and lysozyme-345 (345 bp) promoters as templates. Primers with specific mutation sites were designed for each mutated promoter and listed in Table S1. There are three and four predicted Fkh-binding sites in moricin-242 and lysozyme-345 promoters, respectively. Primers Lyz-D3-Fkh-1, Lyz-D3-Fkh-2, Lyz-D3-Fkh-3 and Lyz-D3-Fkh-4 (Table S1) were used to generate mutations of Fkh-binding site 1, 2, 3 and 4, respectively, in lysozyme-345 promoter. Whereas primers MPAE-Fkh-1, MPAE-Fkh-2 and MPAE-Fkh-3 were used to generate mutations of Fkh-binding site 1, 2 and 3, respectively, in moricin-242 promoter. To pre-screen positive colonies prior to DNA sequencing, restriction enzyme cleavage sites of *Eco*R I, *Nde* I and *Bam* HI were engineered in the mutant Fkh-binding site 1, 2 and 3 of the moricin-242 promoter, respectively. To generate Mor^mut-1&2^, Mor^mut-1&3^, Mor^mut-2&3^ and Mor^mut-1,2&3^ mutant promoters, site-directed mutagenesis was performed using the mutant promoter as the template with the second pairs of primers. For example, to generate Mor^mut-1&2^ promoter, Mor^mut-1^ was used as the template with MPAE-Fkh-2 primers, and to obtain Mor^mut-1,2&3^ promoter, Mor^mut-1&2^ was used as the template with MPAE-Fkh-3 primers. PCR program was 95 °C for 3 min, and then 17 cycles of 95 °C for 1 min, 55 °C for 2 min, 68 °C for 15 min, followed by a final extension of 68 °C for 30 min. The PCR products were recovered, digested with *Dpn* I, and then transformed into competent *E. coli* XL1 Blue cells. The mutant reporter plasmids were then purified and sequenced by an Applied Biosystems 3730 DNA Analyzer in the DNA Sequencing and Genotyping Facility at University of Missouri – Kansas City, and used for transient transfection in S2 cells.

### Dual-luciferase reporter assays

For DNA transfection, S2 cells were placed overnight to 70% confluence prior to transfection in serum-free medium (SH30278.01, Hyclone). GenCarrier-1^TM^ transfection reagent (#31-00110, Epoch Biolabs) was used for transient transfection according to the manufacturer’s instructions. After overnight transfection, S2 cells were centrifuged and resuspended in complete growth medium to induce protein expression for 48 h. Protein expression in cell culture media and cell extracts were analyzed by immunoblotting.

Dual-luciferase reporter assays in S2 cells were performed in 96-well culture plates with recombinant pAC5.1/V5-His A expression plasmid (0.3 μg), pGL3B (empty vector) or different pGL3B firefly luciferase reporter plasmids from the promoters of *M. sexta* moricin, lysozyme, cecropin, defensin-1, defensin-2, defensin-3, attacin-1, and attacin-2 (See Fig. S1 for the promoter sequences), as well as several mutant moricin or lysozyme promoters (0.15 μg), and renilla luciferase reporter plasmid (0.015 μg) (as an internal standard) (pRL-TK, Promega) as described previously^20^. Firefly luciferase and renilla luciferase activities were measured at 48 h after protein expression using the Dual-Luciferase Reporter Assay System (E1980, Promega) in the GloMax^®^ Multi Microplate Luminometer (Promega). Relative luciferase activity (RLA) from S2 cells co-transfected with empty pAC5.1/V5-His A and pGL3B (empty reporter vector) was used as the calibrator. Relative luciferase activity (RLA) was obtained as the ratio of firefly luciferase activity to renilla luciferase activity^70^. These assays were performed in quadruplet and three independent experiments were repeated.

### Immunoblotting analysis and Co-immunoprecipitation (Co-IP) assay

For immunoblotting analysis, cell extracts from S2 cells (2 × 10^6^ cells/well) expressing MsFkh, MsRel2-RHD and MsDorsal-RHD proteins were prepared as described previously^12^. Cell culture media (10 μl each) and cell extracts (10 μl each, equivalent to ~5 × 10^4^ cells) were separated on 10%, 12%, or 15% SDS-PAGE and proteins were transferred to nitrocellulose membranes (162-0097, Bio-Rad). Anti-Flag M2 antibody (F-1804, Sigma-Aldrich, 1:5000 dilution) and anti-V5 antibody (V-8012, Sigma-Aldrich, 1:5000 dilution) were used as primary antibodies, horseradish peroxidase-conjugate anti-mouse antibody (SC-2005, Santa Cruz Biotechnology, 1:10,000) was used as secondary antibody for chemiluminescence using ECL Chemiluminescence Detection Kit (RPN2134, GE Healthcare), and membranes were exposed to films and scanned by Typhoon FLA7000 (GE healthcare), while alkaline phosphatase (AP)-conjugate anti-mouse antibody (A4312, Sigma-Aldrich, 1:10,000) was used as secondary antibody for color development using AP-conjugate color development Kit (#170-6432, Bio-Rad).

Co-immunoprecipitation (Co-IP) assays were performed using cell extracts from S2 cells overexpressing MsFkh, MsRel2-RHD and MsDorsal-RHD proteins. Cell extracts were mixed and Co-IP was performed as described previously^12^. Proteins immunoprecipitated with anti-Flag M2 or anti-V5 primary antibody were captured by protein G Sepharose pre-swollen beads (#17-0618-01, GE Healthcare). Captured proteins were eluted with 30 μl of sample buffer mixed with 0.1% bromphenol blue, heated to 95 °C for 3 minutes, centrifuged at 12,000 × g for 1 min, and the supernatants were loaded onto SDS-PAGE for immunoblotting analysis as described previously^12^.

### Data Analysis

All the experiments were performed in 3–4 replicates and repeated with three independent biological samples. The means of a typical set of data were used to prepare the figures by GraphPad Prism (GraphPad, San Diego, CA). Statistical significance was calculated by one way ANOVA followed by a Tukey’s multiple comparison tests using GraphPad Prism for comparisons of *MsFkh* mRNA in different tissues, or *MsFkh* mRNA in hemocytes, fat body or midgut by different treatments (Fig. 3), expression levels of *D. melanogaster* Fox genes in S2 cells (Fig. 4A), or relative luciferase activity across different promoters by overexpression of DmFkh or MsFkh (Figs. 4, 5 and 7), and identical letters are not significant difference (p > 0.05) while different letters indicate significant difference (p < 0.05). The significance of difference was also determined by an unpaired t-test with the GraphpadInStat software (*p < 0.05; **p < 0.01) to compare the activity of a promoter stimulated by different transcription factors.

## Electronic supplementary material

Supplementary Info
