# Supplementary material for: Transcription Factor Forkhead Regulates Expression of Antimicrobial Peptides in the Tobacco Hornworm, Manduca sexta
Source: Sci Rep. 2017 Jun 2;7:2688. doi: 10.1038/s41598-017-02830-w (PMC5457402; doi:10.1038/s41598-017-02830-w)
Supplement: Supplementary file 1 — Supplementary Info [file 41598_2017_2830_MOESM1_ESM.doc]

**Supplementary Information**

Transcription Factor Forkhead Regulates Expression of

Antimicrobial Peptides in the Tobacco Hornworm, *Manduca sexta*

Xue Zhong^1¶^, Munmun Chowdhury^1¶^, Chun-Feng Li^1,2^, Xiao-Qiang Yu^1^*

^1^ Division of Molecular Biology and Biochemistry, School of Biological Sciences, University of Missouri – Kansas City, Kansas City, MO 64110, USA.

^2^ State Key Laboratory of Silkworm Genome Biology, Southwest University, Chongqing 400716, China

* Correspondence should be addressed to:

Xiao-Qiang Yu, PhD

Division of Molecular Biology and Biochemistry

School of Biological Sciences

University of Missouri-Kansas City

Kansas City, MO 64110

Telephone: (816)-235-6379

Fax: (816)-235-1503

Email: [Yux@umkc.edu](mailto:Yux@umkc.edu)

^¶^: These authors contributed equally to this work.

**This file includes:**

Fig. S1, Fig. S2 and Table S1.

**Fig. S1. *M. sexta* AMP gene promoter sequences.** The followings are nucleotide sequences of *M. sexta* lysozyme, cecropin, attacin-1, attacin-2, defensin-1, defensin-2 and defensin-3 promoter. Fkh-binding motif (AAACA) in the sense strand is underlined, whereas Fkh-binding motif (TGTTT) in the anti-sense strand is dotted underlined. NF-κB binding site is double underlined, while translation initiation site ATG is boxed. In the lysozyme promoter, the predicted transcription initiation site **A** is in bold and boxed. The upstream positions of Fkh-binding site and NF-κB binding site are based on the predicted transcription initiation site (in lysozyme promoter) or the translation initiation site ATG (most promoters).

**Lysozyme:** 1203 bp upstream of the predicted transcription initiation site

TAAACTAATATTGGGACTTTCTTGCCAGTATCAACCTGGAATTTGTGCTCGATATGGCGATAGGCTCGCCGCCTATCACAGCATGAGACTGAACACAAGCGCAAAGTGGGTGCTCTGTTTGTACGTCTGCCTTAACCTTCGGGGATAAAGTCGATATGTGATTGTTTTTTTTTACTAATGATAAGTAATGTTTATGTTATAGAGTTAGGTATCATTATGTAAGTATACATACACATGGACACTATGCTGCGAAAGCACAGGCTTGATTTTCATTTACGTGAAGTACTTACTATGTAACAGATAATTTTTAACGGGGACTATATCAGCAGCAGTTTCTCCAGAATGACATCACGGATCTAAAACCATTCAACTAAATATCGAATATTATATAATCGAGCAAGGGTTGGGCTCTGCGAAGCTTAGAGCTGAGAGAGCTCACCCCACGCTGTTGAATTTGCAAATATGAAATTTTAATGACATAGAAGACTTTTAGTCTGTTAGTTGTGGCCGGGAGAACGATTATTTCCGTTATAAAACGCATATTACCTATTTATATGTTTATTTTTGGCAAATTATATTTGAAACAACTTTTATTTTCAGCTCTTGTAAGGTATGGTATAGGTATTTTAGGTTTTATGTGTACATCGTTATTTTTTTAGAAGATACTATATTGACTAGTTACTGCTGGCTCACTATTAGGAACAATATATGTTATGCATTCTTATAATTATATTTATCACATGATTCTATGCTATATCTTAAGATGAATAAAGGGGTAACCATGGATTATTGTTCTTTCTATTTTTATAAATATGAGAATTTAAAATCTCATTTTTCCAATATAAATAAAAAATACCATAGAACCTTGTGCTAGTAAGCCTACGATATTTTAAAACATAATTGATTTAATCATTAGCATGTATTCTTTTACAGTGTAAGTTAGGTACTAATTAAAAGATTATTAATTAATAAGTTACTAGTCCATAGCTACAGTATTTAAAAACAAACAAAGACATTAATGTAATCTGGATAATACGCTTTCTGTGTAAAACCAAATCCCAACACTAATGTTATTAACAAAGCGCATCACTGAAACATAAACAAACAGATACGCAGGAACCCCCTTTTACAATACACTGCAATACGAGGACAATAATCTATCTATGAGCGCGTATAAAAGCGATCCATTGCGTTCAATTACTC**A**TACGGTTCACAGTCTTGTTGCCGACGACTTCTCAATC

**Cecropin:** 873 bp upstream of ATG

AAAGATTTTTCGAGATAAAAACCCAACGGGTTATATCCATCTAACCTTTTATTAGATACGGACAAACTACATCAGGTTTTTTCTTTGTTTATGGGTACCTAAATGTAGGTATATTTAGCTAGCTATGTGAGGTCCTATATAATAGCGTGTCATTTACTGGAAGCAGTTCCATACTTACGGCTAATATTGAGCAGAAAAATGCATCACTTAATGAACGAGAATCGTATCCGAGACCTCAAGATGGCAATGTTATTACAATACGATTACGTCACCGAAGCAGTTTTTATATTTCGTAAATCATACAAACACATTTTACGGCATTGTCCCTTAAGGGGTTGGCAGAGATGCAACTAGGGCAACTATTTTCCGCCTGTGTATTGCGTCCCATGATATGATAGATGAGCCTATTGCAATATCGGGTACAAATTTCTAATTTCAAACTGATAGTGAGTAGAAAAATTCAATATCACTTGGATCGATCGAGGTTTCGAACCCAAAACGGAAAACCGGCAATGGAACTACGCCGCAGAGATAGTTCCTATATTTTATATCTTTTATTATTATGTTATTTTGCAAACCGCTGCTGCGTGCAGTGATGGGATTTCGGGAAGTACCGTGTATCAAGCGTGTGTGATACTGTGAACTCTTTGACATAACAAAGAGCATCAAAGCGTTCTGTCTGAACGCTGCGTCGTCAATAGCCCTACTGATTAGCAAATAAGGATTATAATTCCATAAACGAAGTGCATTTGTCTTTGTTAACTAACTGTATAGCCATTTTCCCCAAGTGAAGGCGTATAAATAGGGAATTTCTTTAATGAGGGTATCATTAAGCATTTTGTTTTACACTCATCAACAACACAATATAACAAAATG

**Attacin-1:** 387 bp upstream of ATG

AATCCACCGCAACGTAGGGAATTGGCAATGATCTGAGGTTACAATAACATTATTATTTACTTGTTTAAGTTATGGGGATATCATTTATTATTTTTGTTTGATGTATGGGATTTTATACTCGGAATGAATTATTAATGTAAATTTGTATTGATTGTGGTAGTTATCAGGCCGATTATTATTTTATCGTGCGAATAAGACAAAAGGGTCAAAAGTTAAAGCCAGAAAATGATCAAAATGCTTTCAATTTTATGGTTTCTCGAGAATTCCCACGCCGGCACGTATAAGGGGCTGCACTTCTTATCAATACTACATGCGCTGCGACGTATATAAACTGAGACGCAGGCGAAAGCGACATCACTCATCGTCGTGACCCGAGATCGATACATCATG

**Attacin-2:** 2517 bp upstream of ATG

ATCTCCATCCCCAGCCCGAGAATATCCACAATGCGAAAGGCGTTGCGATTTTGTTAATAATAAAACTGGGTTGGGAGTATTTTTGGTCCTCGGACAAGCTCACTGACTGTACCTAACGTCACAGCTATGCTGGTATTTCTGTGAGACGTTATAAACTCTGTAGCAGCGTCCAATTTTTGTTATAAAAAACGACTGCCACGTAGAACAAAAATATACAAGTCATGCCTTCAGGCTTTTCATTATGCAAAGGCAAAAAATGTAGGTATACAAAGTAAAAACATAGCTTCATTTCCGATATATATTCCAAAAAAGCAGGTCGGTCTAAAATAAAATACTTTATTTATCACGTAGGCGAACTAAGTTGCACTTATGATACGTCAAAACAATGTATAAGAATAATTATTATTGTTTCTAAACAACTATTAAAATTACTTATATTACTATGGACATTTCAATTAGGGATTAAATTTATATTGAAAACATGTTACAAACCGAAGTAACCAGCTCGGTCCGGCAGGTGGTGGGAAATAAAAGGATAACGCGTCGCGATCCTCACCGGCGAGGGCATGAGCCCTAACCTAGCTAACCTACCCGCCTTTGACTAGCTACCTAAGTGATGACTACAAGAAGAAGAAAGACTGAAAGAAACTTTTGGTCTATAGTAGTTTTAATTAACATTTTTGAATCAATCTCACATGAAGTTGCATCGCAGCATGAGTATTTTTTTGAGTTCTTATTTTTTCAAACATGATGTTTTTTATTATTTTCTTCTCACCGTCCTTGATGCCACTTCAATTTAAAACGCGTTTTCACAACCTATCCGAAGTTAACTTACTCTAGAGCATCTAAATCAAAACTTGGAATAATAAAAATATTATTAGACCTACGAATGGAACCCAGAACCTAACCATAAAACATACGCTGCCACAAGACCAAGGAGGTGACATAAACGTTATTACATAAAGACGTTTAAATGGCGTATAGAGACGATAACAGTGTTTACAGTAACAACGGTTAATTAATTGCGGTTTAGGTCAAAGTTAACCGATACACTATCGCTGGGGTTTCGTCAGTCCAGACGACGCGGATACTAATTTACAAGATGTGCCATTCTAGCAAAGCGATATTGGATTTCTCTACTCAGTGTCAGCCAGTGACTTTTAAGAAAAAAAATATTGGAAGGATCGGTAGACCTTTAACCTCTCAAATGTCAATCAAACTGCGGCGCTAACATTGGATATAAACAGATTTACAAAAAATAGAGTCCTATGTATTGGAACACATTGATGTTCAGTATTTTAGGTATTTCGTTTAGTTACTTAAATTAACGAATTCAAGATATTAAGTCACTTAATCTGCACCACAATACCAAATTAAGAGCTAGGATGGTGCCAGATTTTTTATATAGTCTTTGCACAACGATAAATGAACACACTTTTGAAAACCCGTCTAAATGGTGCGGTCCAACGAGCCGTCTTTAGATCAGACCGCTTTATAAGTCATAAATTTCAGGAGTCTGGAATTTGTGACTGCTATGGCGGTAAACTCGCTCCCTATCCTGAGATGAAAAACACTTGGTGAAAAGTGGGTGCTCTGCTTGCACCTCTGCCTACCCTTACGGGAAAGGGCGTTAAGTATGTGTTTGTTTGTGCTAAAGGGTGCTTTGACATTTCAGTAAATTTGTGATTTATAAGGTATGTTTAGATTTGTATGTATCATGGAAATGGATGCGCGGATCATTTGAGTTTAGTTATTATGAATAATGAAAAAACAAGGGACATCAAATTCGTATATTTTTTTATAGAATTATGTTGACATCTTAAATGAACACACACATGTGATGCCTTACACTAAATGGAATAAAGAATTCCCCTTTTTTTCTTTAAACACAAAGCAAAAAAATATTTTTTATATTAAAAGGATATTTTGCTAATTATAATTGTAGGTTTTAAAAAACTCCTTAGGGAAAATCATGATAAAACACATTATATTAATTCGTGATATACTTACAACAATAATATTTTGATAGCAAGATAATATTATAGGCAAAATATATTTCTTTATTTTAGAAACATTTTTTTATCACAGATGATAGCTTTACACCACTTTCTTTCAACCAGTTTTCCAACAATGATAAGGTCCATGTTGCGCGGGCGAGTAGTCGGTAAGGAACGAGACGCTTCGGGTTCACATCCAGAACTGTTTTTTTTTATTTGAATTCCAAATGTTTGTTTGAGTTTTGAGTGTTGTGACAGCGAATTCAATAATGGTTACAAACTATCAATATGCCCAGTAAATTATAAAGGTGTTTCATTTTATTAAATTAATAAAACTTAATTATGTATTTGACACATCCAGGCTGACCTACATCTAATATTATTCCCCGACCTATAACACTCTCATTTCACACACATACGAATTCGCGATTACGGGGAAGAACTCAAATCTTCCAATTTCGTATATAAACTCAACTCATTTCTACCAAGCCATCACTCGCTAGTTCGCAACAGCACCAATATG

**Defensin-1:** 614 bp upstream of ATG

ACACCAGTATTATAATGTTATATTTGAACTTAGTTGTTTTTAAACTGACTAAACAGCTTGAGTTTAGTATAATTATATAGTAAATATGTTATAATACAAAATTAATGGAACATAAATATGTGTGAAATCCACAAATACGGACACTGACGCCTTTCATCCCGGAAAGTCTAGGCAGAGGCGTATTTCATCCTGTGATGTGATAGGGGGCGAGCCTATCGCCATGTCGTGTTATTACTTTATATTATAAATTCAATAGCACTGATTAGGGATATGAATATGCCTCAAGCATTCAAATACAAGAATATCAAATCCATTGTAATACAAGGTATTATCTATTACAATGGCTAATGATAAGCTATAATTATATTAAAATAATATTCCATAATTTATTAAAGAGTAAGGAATTGACTCTGAAGTTCTGAGTATATGCGCTGATTACATGTTTAGTAATAATGAGTCACTACTCATTCTCGGTGTAGCGGTACCTAATTACGCATTCGATAACAGAATAACTTAGAGTCCGCACGTATATATAGTACTGCTCCCGTTGACCTCTACTTATTTCTATTCGGTTCGGTGCCACTTCATTGTCTATAAAACAGATTGAAATAAAAATG

**Defensin-2:** 554 bp upstream of ATG

AAATAAAATGGTGATTATACGTTATAATGATTGATATTTGTTTCTAATTATTTCTACTAATTCAATAGCAGCGATCAAATAATCTATATACTCGTAACACATATTTTTTTTCCTGTACCAGTAACCTGTTTGTCTAAAAGTCGACCTTATAAGAGATTCATAAGTATTTCACCTCAATTTTTTTTTTATTAAAACCATTGTAAATGATTAAAAATCTATAATAAGCGTAAATAGCGATGATAAATAAAAAATGTACAATAAGGTTAATTGGTAATATTGCGAAGTTACACGACGTAAAGTACTGCGTCGAAAGGTTCAAGAACCTGGAGTTTCCTTGTTTTTTTTTTAGTCATTTATATTCTGCTCATACGGCCCATTGTTCATCTTAATTCTCGGTATACGAATGTTTTGCTGCATTAACCTACATCCAAACCTATATATACCGAGACTGACGGCTTTTAACACAGTTGGGATTTGTATCTCCTCACTGAAGCATCACTAGTGTTTGACAGTAGTTTTATAATAATCATCAAAAGCTCAATGTTGTTGTTGGTATG

**Defensin-3:** 731 bp upstream of ATG

GTTATGAATCAATACACGTTACATTTAACGTATACGCTAACTATGGTAGAAGCCACAACTTAACTTAACTTAACTTACATTTGTCTTAAAATGTGATAGAGTGTACTTATGTATGTATCCAGAAAGTATTTTGAAGTCATTCTTGGTTTTAGATACTCTTAAGTTAAATTATCATCATATATCATGCAGTAGTCCCGTGCGACACTCCGCTACATTTTAATTTTCCCTTCCTGTGAAGAGGGTCAGATATGACGTTTATCTTGGCAGAAAGGGCCTGCGAATCTGCCTTTTTTGTTTATTAATGTTTTTTTGCTTCAAGTTTAGCCGTTATAGGGGTCGATGCAACACGTTCTAATTTCGAGGAAAAAGAAATCTTATTACCTTCAATGTCTCAAAAAAAGACAGCAGACGTATATTGATGAAAAAGAGCGTAAGTAGCATCTAAATATAATTTCATCATCTAGTTTAACTTAATAAAACAATTTGATTCTTAATATTGCGCCCTGTATAAGGTACCTATAATTTATATTGAATTTAGTCTATAAATAAACAACGACAGAGTTACAGAGCTTTTAACTTTATTATAATACGTTACGACACAGTTCTGTCTGCTATTGATCAAGACTCTAATGATTAAAGTTATGATCCACTGGGTTTGTATGCACGACATTCAAAAATCATTGCTAAGTTTTTACTTGTGCCTGTAGGACGTCTCCTTTTTGTAAACCAAAATG


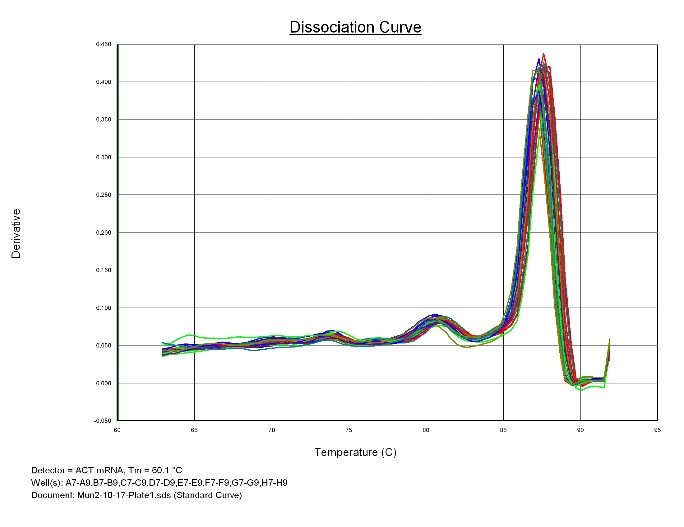

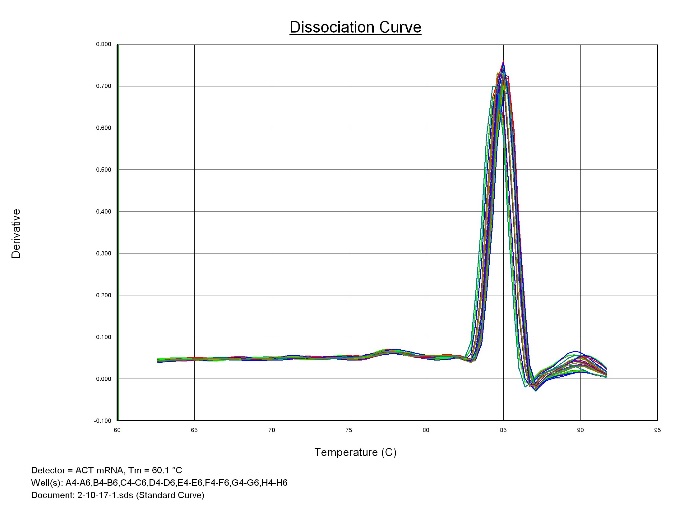

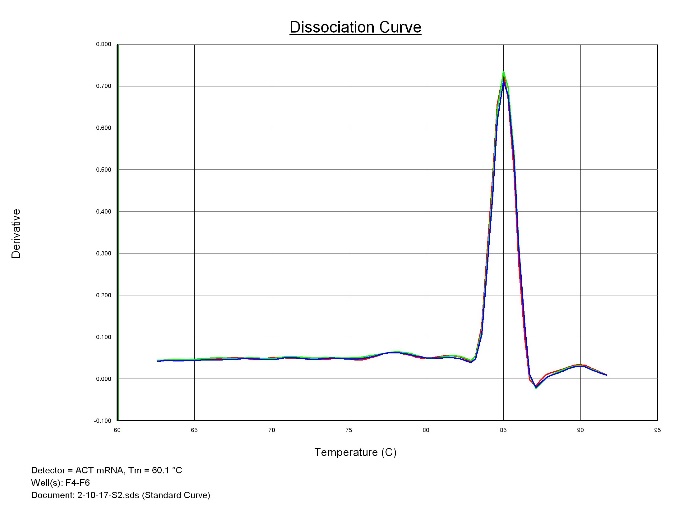

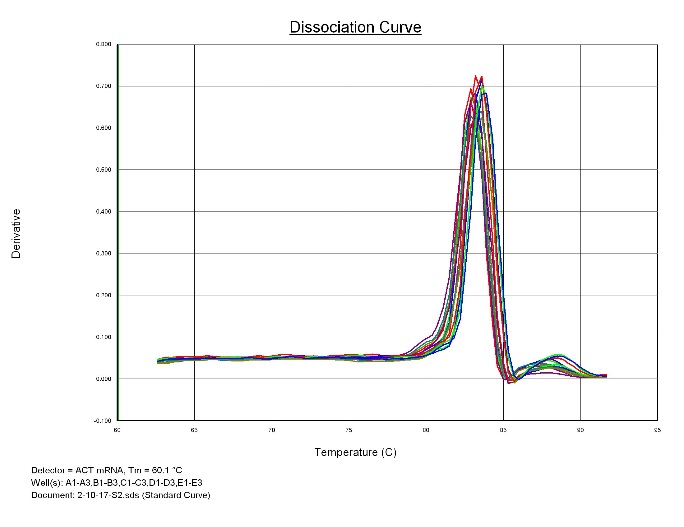


**A**

**B**

**C**

**D**

***M. sexta***

***rpS3***

***M. sexta***

***Fkh***

***Drosophila***

***rp49***

***Drosophila***

***Fox genes***

**Fig. S2. Dissociation curves of *M. sexta* *rpS3* and *MsFkh*, as well as *D. melanogaster* *rp49* and Fox genes from real-time PCR.**

Table 1. PCR primers used in this study

| **Primers** | **Forward Primer (5’-3’)** | **Primers** | **Reverse Primer (5’-3’)** | |
| --- | --- | --- | --- | --- |
| **Cloning** |  |  |  | |
| MsFkh-F-Kpn I | **acggGGTACCATGATCTCGCAGAAGTTATCGTACGGCG** | MsFkh-R-Not I | **AagaatgcggccgcgaatgcggccgCttCAAGGGCGGCTGC** | |
| DmFkh-F-Kpn I | **acggGGTACCATGACCAGACCACCATTGACATCATGCAGAAGCTCTACGCGG** | DmFkh-R-Not I | **aagaatgcggccgcTTACAAGCTCGTGGTTCCGGCGG** | |
| Lyz-D3-Fkh-1-N | **CTACGATATTTTAGCGGCCGCTAATTGATTTAATCATTAGC** | Lyz-D3-Fkh-1-C | **AATCAATTAGCGGCCGCTAAAATATCGTAGGCTTACTAGC** | |
| Lyz-D3-Fkh-2-N | **AGTATTTAAAAACGCGGCCGCAAGACATTAATGTAATCTGG** | Lyz-D3-Fkh-2-C | **TAATGTCTTGCGGCCGCGTTTTTAAATACTGTAGCTATGG** | |
| Lyz-D3-Fkh-3-N | **AAGCGCATCACTGGCGGCCGCTAAACAAACAGATACGCAGG** | Lyz-D3-Fkh-3-C | **GTTTGTTTAGCGGCCGCCAGTGATGCGCTTTGTTAATAAC** | |
| Lyz-D3-Fkh-4-N | **CTGAAACATAAACGCGGCCGCGATACGCAGGAACCCCCTTT** | Lyz-D3-Fkh-4-C | **CTGCGTATCGCGGCCGCGTTTATGTTTCAGTGATGCGCTT** | |
| MPAE-Fkh-1-F | **TGTATATGTATAGAATCCTGGCAGATTATAATATGAATG** | MPAE-Fkh-1-R | **ATAATCTGCCAGAATTCTATACATATACATTTTAATTTAAAAG** | |
| MPAE-Fkh-2-F | **TAGGAATAGGTACATATGTCGACTAGTATACGTGTTACG** | MPAE-Fkh-2-R | **GTATACTAGTCGACATATGTACCTATTCCTAATCATTCATAT** | |
| MPAE-Fkh-3-F | **CGCGTTTAATAAGGATCCTAATATAAACGCTTATGACAA** | MPAE-Fkh-3-R | **GCGTTTATATTAGGATCCTTATTAAACGCGACCCGTAACAC** | |
| **Real-time PCR** |  |  | **Amplicon size** | |
| MsFkh-N | **GAGCCAAGCGGGTACGCG** | MsFkh-C | **GGTACAGGGGCGACTGGT** | 151 bp |
| DmFkh-N | **ATGTCGGCAGCGAGTATGTC** | DmFkh-C | **ATGCGTGTAGCTCCTTCTGT** | 150 bp |
| DmFoxK-(L+S)-N | **GCTCACCGCTCCAAGATA** | DmFoxK-(L+S)-R | **TCTGCTGCTGCTGCTGCT** | 150 bp |
| DmFoxK-(L)-N | **CAGCATCGTTGTGGCCCC** | DmFoxK-(L)-R | **TTGGCAGCGCTTATTGTA** | 150 bp |
| Jumu-N | **ATGCCAACGGAAACCAGG** | Jumu-R | **TTCGAGATAATTCGACGC** | 150 bp |
| dFoxO-N | **GGCCACGGTCAACACGAA** | dFoxO-R | **TGCTGCTGCTGCTGCTGT** | 150 bp |
| rp49-N (*Drosophila*) | **GCCCAAGGGTATCGACAACA** | rp49-C | **ACCTCCAGCTCGCGCACGTT** | 150 bp |
| rpS3-N (*M. sexta*) | **CCCGTGGGACCAACAGGG** | rpS3-C | **GCGGCGACCGGCTGCGGC** | 150 bp |
